# Public awareness of malaria in the middle stage of national malaria elimination programme. A cross-sectional survey in rural areas of malaria-endemic counties, China

**DOI:** 10.1186/s12936-016-1428-x

**Published:** 2016-07-19

**Authors:** Shangfeng Tang, Lu Ji, Tao Hu, Ruoxi Wang, Hang Fu, Tian Shao, Chunyan Liu, Piaopiao Shao, Zhe He, Gang Li, Zhanchun Feng

**Affiliations:** School of Medicine and Health Management, Tongji Medical College, Huazhong University of Science & Technology, Wuhan, Hubei People’s Republic of China; Cancer Center, Sun Yat-sen University, Guangzhou, Guangdong People’s Republic of China; Bureau of Disease Prevention and Control, National Health and Family, Beijing, China; University of Nottingham, Nottingham, UK

**Keywords:** Malaria, Knowledge, Awareness, Elimination, Malaria health promotion, China

## Abstract

**Background:**

Remarkable progress in the elimination of malaria has been achieved by the Chinese government in the past 5 years. However, imported cases have increased rapidly, and it is a critical threat to the national malaria elimination programme. This study aims to investigate the current status of the public awareness of malaria in the middle stage of the national malaria elimination progress.

**Methods:**

A cross-sectional survey with multi-stage stratified randomized sampling was undertaken between June 2015 and March 2016. A total of 1321 residents from nine malaria-endemic counties, 27 townships and 81 villages were interviewed using a structured questionnaire.

**Results:**

The results showed 51.6 % of the respondents had sufficient malaria knowledge. The malaria awareness of the public in type I counties was better than that in type II, whereas that in type III was the lowest. Approximately 74.9 % of the respondents were aware of at least one form of prevention of malaria, and 85.2 % of them would seek treatment when suffering from malaria. However, the awareness of fever, chills, sweating as common symptoms of malaria were 53.4, 56.2 and 31.6 %, respectively. The level of malaria awareness of the at-risk population was similar to that of the general population, it seemingly increased along with age and declined with the distance away from township hospitals.

**Conclusion:**

The public awareness of malaria needs to improve continuously. Health education campaigns should focus on basic malaria knowledge and cover target populations. The multi-sectoral or even international collaboration should be further intensified. Careful planning is required to ensure that scattered villages are incorporated into the malaria health promotion system to sustain elimination.

## Background

Malaria is one of the major public health problem across the world. The World Health Organization (WHO) has reported 214 million new cases of malaria and 438,000 deaths in 2015 [1]. Historically, China was a malaria-endemic country. The large-scale outbreaks of malaria which occurred in the 1960s and 1970s severely influenced public health and socioeconomic development [2], especially in central China [3]. The combination of human behaviours and livestock reduction was explored as an important factor contributing to some of these outbreaks [4, 5]. The number of malaria cases decreased significantly [6], and the incidence of malaria has declined from a peak of 2961/100,000 in 1970 to 0.1/100,000 in 2014 [2, 7]. The last decade has witnessed a decline of malaria prevalence in China and the government has set the target to eliminate malaria by 2020 [7].

The roadmap for elimination was developed alongside the initiation of the national malaria elimination programme (NMEP) [8]. All malaria endemic counties in China were divided into three types according to annual incidences [9]. The endemic counties, ones where autochthonous infections had been detected continuously from 2006 to 2008 and the annual incidence had been no less than 1/10,000, were categorized as type I. The ones where autochthonous infections had been detected continuously and the annual incidence had been lower than 1/10,000 at least in 1 year were classified as type II [10, 11]. Other counties with no autochthonous malaria cases during these 3 years were defined as type III. Since the initiation of the NMEP in 2010, China has achieved a remarkable autochthonous malaria-eliminating progress, and malaria map had shrunk dramatically [12]. However, given that imported cases have increased rapidly in recent years [13], risks of recrudescence and transmission need to be taken into account urgently by counties that had already passed the sub-national elimination assessment [14]. The malaria transmission in infection hotspot is important predictor infection of malaria in the future [15]. Therefore, the high at-risk populations were defined as returnees from the malaria epidemic area, such as Africa and Southeast Asia, and residents living in malaria foci, who require greater consideration.

Health education and health promotion was the indispensable part that contributed to the elimination of malaria [16, 17]. As in previous studies, malaria awareness campaigns played an important role in health promotion by increasing the level of knowledge regarding malaria and improving health-related behaviours, as well as promoting inter-sectoral collaboration and social support [10]. In China’s plan for malaria elimination, public awareness of malaria has been set as an essential index to evaluate elimination progress [9], and it aims to cover various populations and increase the level of awareness of malaria among residents in type I and type II counties to 80 % by 2015. In a recent review of research activities on malaria in China, most malaria studies have been undertaken by Chinese Center for Disease Control and Prevention (CDC) or its affiliated units [18], and independent third parties have been involved in investigations related malaria issues. In order to achieve the final goal of malaria elimination by 2020, this study was carried out in the middle stage of NMEP to investigate the current status of public awareness of malaria, contribute to an adjustment of the strategies to improve public awareness of malaria in the next elimination phase.

## Methods

### Data collection and sampling

This cross-sectional survey with stratified multiple-stage sampling across malaria-endemic counties, townships, villages and populations was conducted in rural areas of Guangxi autonomous region, Chongqing municipality, Anhui and Hubei provinces between June 2015 and March 2016. Similar to previous stratified methods, a four-step sampling procedure was employed [19–21]. Firstly, all malaria-endemic counties from those areas were divided into three types (I, II, III), and three malaria-endemic counties were randomly selected from each type in rural regions (i.e., 3 × 3 = 9 counties). Secondly, based on the malaria-endemic status, all townships in sample counties were divided into three groups, and one was selected randomly from each group (i.e., 3 × 3 × 3 = 27 townships). Thirdly, one village was randomly selected from each of the three groups that were classified by the distance to township hospitals (i.e., 3 × 3 × 3 × 3 = 81 villages). Lastly, twenty residents aged above 15 years old were randomly selected from each sample village. By excluding implausible or missing data, 1321 participants were finally enrolled in this investigation. Based on the literature review, a structured questionnaire was designed to measure the basic understanding and knowledge of malaria. Participants were interviewed face-to-face by trained medical postgraduates from the School of Medicine and Health Management.

### Data analysis

The database was established by EpiData Info version 3.1 (Atlanta, Georgia, USA) and data were double inputted for quality control during the entry process. Predictive Analytics Software statistics 12.0 (SPSS, Chicago, IL, USA) was performed for data analysis. According to previous studies, the levels of knowledge with accurate rates above 80 %, 60–79 %, 0–59 %, and 0 were considered excellent, good, poor and very poor, respectively [10, 22]. The level of knowledge regarding malaria for each subgroup was calculated based on the scores of 13 malaria questions, and descriptive statistics were performed for summarizing basic information. One-sample *T* test was employed to test the differential means of malaria knowledge across dichotomized variable, and the means across other demographic covariates were tested by one-way analysis of variance. Chi square test was applied to test the differences in the correct rates of the responses across three types of endemic malaria counties. The p value of 0.05 or less with double-sided was termed to be statistically significant.

## Results

### Demographic characteristic of study population

The mean age of participants was 44.78 years, (SD 17.50) and more details were shown in Table 1. Most participants were the Han nationality (65.5 %), and married (87.2 %), but the high at-risk population accounted for only 12.8 % of the sample population. In accordance with the Chinese government’s effort to improve the accessibility of health care services for rural residents, most villagers lived close to village clinics (64.3 %).

**Table 1:** Background, characteristics and scores

Socio-demographic characteristics	n	%	Basic malaria understanding				Basic malaria knowledge				Malaria knowledge			
			Mean	SD	T/F	P	Mean	SD	T/F	P	Mean	SD	T/F	P
Gender					3.5	<0.01			4.1	<0.01			4.2	<0.01
Male	643	48.7	1.94	0.95			5.68	2.78			7.62	3.52		
Female	678	51.3	1.75	1.01			5.05	2.80			6.80	3.58		
Ethnicity					14.8	<0.01			12.8	<0.01			15.0	<0.01
Han	865	65.5	1.92	0.94			5.55	2.74			7.47	3.46		
Minorities	456	34.5	1.70	1.05			4.98	2.88			6.68	3.72		
Age					10.8	<0.01			6.9	<0.01			8.7	<0.01
<29	288	21.7	1.59	1.05			4.73	2.79			6.31	3.62		
30–44	325	24.6	1.80	1.01			5.36	2.84			7.16	3.65		
45–59	394	29.9	1.94	0.93			5.61	2.79			7.54	3.49		
>60	314	23.8	2.00	0.91			5.62	2.73			7.63	3.43		
Education attainment					0.8	0.46			5.6	<0.01			4.4	0.01
Less than 7 years	566	42.9	1.82	0.99			5.15	2.79			6.97	3.56		
7–9 years study	517	39.1	1.83	0.98			5.34	2.84			7.17	3.63		
More than 9 years study	238	18.0	1.91	0.98			5.87	2.69			7.79	3.44		
Occupation					7.2	<0.01			6.5	<0.01			7.2	<0.01
Self-employed worker	219	16.6	2.01	0.93			5.57	2.64			7.58	3.36		
Farmer with earning power	778	58.9	1.88	0.95			5.48	2.81			7.36	3.53		
Non-work	193	14.6	1.57	1.12			4.51	2.90			6.08	3.85		
Worker	52	3.9	1.97	0.79			6.15	2.58			8.13	3.12		
Student	79	6.0	1.59	1.05			5.05	2.74			6.65	3.58		
Number of household member					18.3	<0.01			10.1	<0.01			13.3	<0.01
<3	404	30.6	2.07	0.83			5.77	2.63			7.85	3.21		
3–4	689	52.2	1.80	1.02			5.37	2.84			7.17	3.65		
>4	228	17.2	1.60	1.06			4.72	2.83			6.33	3.70		
Marital status					3.6	0.06			3.5	0.06			4.0	0.05
Unmarried	203	15.3	1.72	1.05			4.99	2.80			6.72	3.64		
Married	1118	84.7	1.87	0.96			5.41	2.76			7.29	3.50		
High at-risk populations					3.0	<0.01			0.5	0.62			1.2	0.23
Yes	169	12.8	2.02	0.82			5.44	2.41			7.47	3.02		
No	1152	87.2	1.82	1.00			5.34	2.86			7.16	3.65		
County					39.8	<0.01			36.3	<0.01			42.0	<0.01
Type 1	404	30.6	2.09	0.74			5.93	2.53			8.01	3.02		
Type 2	515	39.0	1.91	0.97			5.65	2.72			7.56	3.48		
Type 3	402	30.4	1.50	1.12			4.40	2.94			5.91	3.86		
Self-assessment of household income level					1.2	0.31			6.3	<0.01			4.6	0.01
Low	560	42.4	1.83	0.97			5.07	2.72			6.90	3.48		
Middle	639	48.4	1.87	0.98			5.63	2.84			7.50	3.60		
High	122	9.2	1.72	1.08			5.17	2.89			6.89	3.79		
Distance to village clinic (Km)					0.6	0.62			0.2	0.88			0.2	0.92
<1	849	64.3	1.81	1.00			5.30	2.81			7.11	3.60		
1–2	281	21.3	1.88	0.94			5.33	2.75			7.22	3.45		
2–3	126	9.5	1.89	0.98			5.11	2.49			7.00	3.25		
>3	65	4.9	1.75	1.03			5.18	3.05			6.93	3.89		
Distance to township hospital (Km)					6.4	<0.01			3.3	0.02			4.3	<0.01
<1	360	27.2	1.95	0.94			5.57	2.64			7.52	3.36		
1–2	323	24.5	1.92	0.89			5.28	2.63			7.20	3.26		
2–3	235	17.8	1.75	1.07			5.22	2.92			6.97	3.79		
>3	403	30.5	1.68	1.03			4.93	2.89			6.60	3.72		

### Overall average of malaria knowledge

**Fig. 1:**
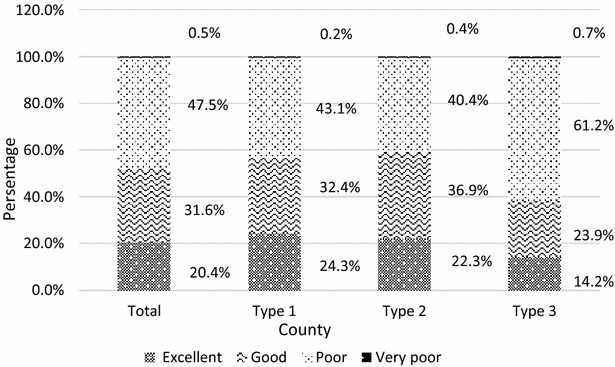
Percentage of residents in term of the scores for knowledge of malaria. The accurate rates above 80, 60–79, 0–59, and 0 % were considered excellent, good, poor, very poor

As shown in Fig. 1, 51.6 % of the respondents achieved an over-60 % correct rate, among whom, 20.4 % had excellent knowledge of malaria. Unfortunately, 47.5 % of the respondents had poor knowledge of malaria. In general, compared with those from type III counties, participants lived in type I and type II counties were more likely to have higher level of knowledge of malaria.

### Differences in malaria knowledge across covariates

In terms of the mean level of knowledge of malaria across the socio-demographic covariates, significant statistical differences were found, and more details were shown in Table 1. Participants with a higher level of knowledge regarding malaria seemed more likely to be males, Han residents, married, with education level and smaller household size, and workers or farmers with earning power. In addition, the high-risk population aforementioned text had a greater awareness of the basic understanding of malaria, but at a very basic level. The mean scores of knowledge were similar between students and farmers without earning power and were lower than others. Most notably, the mean level of knowledge of malaria among residents seemed to increase with age whereas decrease along with the distance away from township hospitals. However, there was no difference found between knowledge levels and subgroups of the distance to village clinics.

### Awareness of basic understanding and basic knowledge

Among these 1321 rural respondents, the overall correct rate regarding the knowledge of malaria was 55.4 %. The correct rate of basic understanding and basic malaria knowledge were 64.4, 53.5 %, respectively. As shown in Table 2, statistical differences were found in the awareness of malaria among residents from different types of malaria-endemic counties. Similar to a previous study [10], the public awareness of malaria in type I counties was greater than that in type II, and that in type III was the lowest. But residents with the greatest awareness of treatment-seeking behaviours were from type III counties.

**Table 2:** Distribution of major malaria knowledge in rural China

Items	Awareness (%)				F	P
	Total	Type 1	Type 2	Type 3		
Overall malaria knowledge	55.4	61.7	58.2	45.4	42.0	<0.01
Part A basic malaria understanding	61.4	69.6	63.8	50.2	39.8	<0.01
Part B basic malaria knowledge	53.5	59.3	56.5	44.0	36.3	<0.01
Major symptoms of malaria	47.1	56.6	49.2	34.9	35.6	<0.01
Causes of malaria	44.7	48.7	47.2	37.4	12.0	<0.01
Prevention knowledge	58.3	66.3	62.6	44.8	34.9	<0.01
Treatment-seeking when suffering from malaria	85.2	77.7	88.2	88.8	13.0	<0.01

### Details regarding the awareness of malaria

The details of the results were listed in Table 3. In part A, a high percentage of residents were aware of malaria and considered it as one that could be cured, but only 36.3 % of the respondents were aware of that malaria was an infectious disease. In part B, the percentages of who were aware of major malaria symptoms including fever, chills, and sweating were 53.4, 56.2, and 31.6 %, respectively. The percentages of the respondents who knew at least one symptom were 82.2, 70.9 and 53.5 %, for type I, II and III counties, respectively. In total, 69 % of the respondents identified malaria vectors correctly, but only 49.8 % of the respondents in Type I counties were aware that malaria was transmitted by mosquito bites, which was lower than those in other malaria-endemic counties. 74.9 % of the respondents knew at least one method to prevent malaria, and the percentage of who knew to take medicine against malaria before going to endemic areas and take self-protective measures against mosquito were 54, 54 and 63.3 %, respectively.

**Table 3:** Public awareness of malaria in rural China

Items	Awareness (%)				χ2	P
	Total	Type 1	Type 2	Type 3		
Part A basic malaria understanding						
Aware of malaria	76.3	89.9	78.3	60.2	99.8	<0.01
Malaria is infectious disease	36.3	38.4	37.9	32.1	4.4	0.11
Malaria can be cured	68.7	70.8	75.1	58.2	31.3	<0.01
Part B basic malaria knowledge	53.5	59.3	56.5	44.0		
Major symptoms of malaria attacked						
Fever	53.4	59.4	60.0	39.1	48.1	<0.01
Chills	56.2	64.9	57.3	46.3	28.6	<0.01
Sweating	31.6	45.5	30.3	19.4	64.4	<0.01
Knows at least one symptoms	69.0	82.2	70.9	53.5	79.0	<0.01
Causes of malaria						
Mosquito bites	34.6	49.8	32.8	21.6	71.6	<0.01
Bad food	56.6	56.4	65.2	45.8	34.7	<0.01
Malnutrition	38.2	41.6	38.4	34.3	4.5	0.1
At least one answer is corrected	69.0	74.3	74.6	56.5	42.2	<0.01
Prevention knowledge						
Taking medicine against malaria before going to endemic area	54.0	64.4	56.3	40.8	46.8	<0.01
Faraway from animals attacked by mosquitoes	54.0	54.5	60.8	45.0	22.6	<0.01
Anti-mosquitoes	63.3	68.6	70.7	48.5	54.7	<0.01
Knows at least one prevention	74.9	86.6	78.6	58.5	91.3	<0.01
Treatment-seeking when suffering from malaria	85.2	77.7	88.2	88.8	25.6	<0.01

## Discussion

This study has shown the pubic awareness of malaria in the middle stage of the NMEP, which is unsatisfactory to the demand of elimination in the perspective of health education and promotion. It is a major concern that the level of knowledge regarding malaria still needs to improve in the sample counties. A similar study conducted in 2014 reported that 43.7 % of the Indians had poor knowledge of malaria [23], however, the percentage of respondents with poor knowledge reached 48 % in this study. Compared to the situation at the beginning of the NMEP [10], the percentage of respondents with an over-60 % correct rate has declined from 58.86 to 52.00 %. This could be explained by that subtle differences occurred in measured questions, and residents from type II counties in previous studies were responsible for 82.56 % of the sample population, which contributed a great impact on the high level of knowledge regarding malaria (see Fig. 1). In addition, elimination has allegedly been achieved in the majority of the sample counties. The focus of health staff in daily work may be shifted to the control of other diseases. Lack of staff or passive publicity activities may be the substantial reasons leading to these unsatisfactory results.

Higher public awareness of malaria contributes to malaria prevention and elimination, and it could improve self-protection [24], early treatment-seeking [25], and disease detection [26]. Although some studies from Africa have argued that the practice of protection measures was independent of personal knowledge [27, 28], others have confirmed the impact of sufficient knowledge on behaviours and the effectiveness of the control measures [29], and reduced the probability of malaria infections [23]. The current status of public awareness of malaria in type I and II counties still has much to catch up in order to achieve the objective of 80 % of residents having good malaria knowledge, which was set at the initiate of the NMEP. Poor public awareness may be unresponsive to the potential malaria resurgence [14], the results indicate that more attention should be paid particularly to type III counties, where approximately 61.9 % of the respondents have a poor level of knowledge, which is consistent with the experience from Indonesia [30].

In terms of the basic understanding and knowledge of malaria, a present study also reported that compared to other subgroups, students and the youth aged below 29 had the poorest level of knowledge regarding malaria and even lower than that at the beginning of the NMEP [11]. The main reason for this might be the selection bias that some of them still left their houses for hanging out, travelling or working. Additionally, the level of knowledge is similar to that found in a study of a survey regarding the knowledge of malaria among high school students in Thailand [31], but it is lower than that in Tanzania a decade ago [32]. In addition, the average level of knowledge regarding malaria seemed to rise along with the distance to township hospitals. In line with a similar study conducted in Iran, township hospitals were the most important source to disseminate information related to malaria [33], and people who live close to township hospitals might acquire more knowledge due to more interaction with the health staff. Simultaneously, a longer distance to township hospitals might result in less frequent visits given the cost and loss of work time [23]. However, no difference was found in the distance to village clinics. It suggests that as the scattered village clinics are close to inhabited regions, village doctors in those clinics should be involved into the malaria health promotion system.

Interestingly, the knowledge of malaria of the at-risk population as aforementioned in the text is similar to that of the general population. In accordance with previous studies [34], it is far from satisfactory. Therefore, it is vital to improve the knowledge regarding malaria of the at-risk population to enhance their self-protective awareness and reduce any potential recrudescence risk. Nevertheless, the health education campaign covering migrant population involves various departments, such as Public Security, Commerce, Health and Family Commission, Quarantine Bureau, and Social Media. (unclear meaning) [10]. This may often be practically intractable, which requires an improvement in multi-sectoral cooperation. Simultaneously, in the context of China’s “Belt and Road Initiative” and its aid programmes in African countries [35], malaria cases, most of which were imported from Africa and Asia [36], have increased rapidly, which becomes as a critical potential challenge to the elimination programmes [13, 37]. Meanwhile, further communication on malaria education could be developed between China and countries in Africa and Asia. However, even if this may be intuitively appealing, this is often practically intractable for the populations at risk, because cooperation in health education between different sectors is usually complicated.

Findings of this study also show that the public awareness of basic malaria knowledge is poor, especially regarding symptoms of malaria, and vectors involved in the transmission of malaria. Only one-third of the rural respondents considered malaria as an infectious disease, and the proportion is lower than that in Vanuatu [38]. This indicates that more than half of the residents just hold a simple understanding that may lead to residents’ low level of alertness of malaria infection and a lack of self-protection. This study also showed the negative results of the public awareness of malaria, and it needed to improve continuously. Although more than two-third of the respondents knew at least one main symptoms of malaria: fever and chill were widely termed as common symptoms of malaria, however, the public awareness was still lower than that in other Asia–Pacific counties [39, 40] and Iran [41]. Moreover, only one-third of them know that the mosquito is the vector of malaria transmission and the proportion tend to be much less than that in Myanmar [39], Iran [41], Cambodia [42] and Peninsular Malaysia [43].

Nevertheless, this study also showed that the majority of the respondents knew about malaria prevention. Compared to other studies, public awareness of medication to prevent malaria was higher than that of Ethiopia [44], a country heading towards the pre-elimination phase. The awareness of seeking treatment when suffering from malaria was considerably higher than that reported from Cameroon [45]; this may be due to the large-scale self-medication in that country. Interestingly, people do not know that anti-mosquito measures are an effective method to prevent malaria, but they still use those measures. This may be explained by the fact that mosquitoes have always been considered as one of the most harmful pests by the Chinese government, for carrying pathogens and biting people, and it is rooted in the social environment. It shows that raising the public awareness of malaria among populations is possible, with sustained effective health education and promotional activities over a long time.

### Limitations

There are several limitations in this study. First, the sample size is small. In China, the population of one county may reach approximately 500,000, only 180 residents in rural areas from one counties were enrolled, it may result in a selection bias. The strict sample procedure across four stages was controlled, and the indoor interview was conducted, it can represent the population of sample counties and the awareness of the population. Secondly, the distance to village clinics or township hospitals and the household income are hard to estimate. The data were collected by respondents’ self-reporting, which certainly is subject to individual variances. Hence, the assessment criteria were assumed as the same for every respondent in this study.

## Conclusions

The public awareness of malaria, except the knowledge of prevention and treatment-seeking when suffering from malaria, needs to improve continuously. Health education campaigns should publicize basic malaria knowledge, such as the symptoms, transmission vectors of malaria, and focus on target populations including the youth, students, and residents living in remote areas and the areas that have achieved elimination. Moreover, the multi-sectoral and even international collaboration should be further enhanced in malaria health promotion. The radiation effect of township hospitals seemed to decline with the increase in the distance to township hospitals, and careful planning is required to ensure that scattered villages are incorporated into the malaria knowledge promotion system to sustain elimination.

## Abbreviations

WHO: World Health Organization
NMEP: National Malaria Elimination Programme
SD: standard deviation
CDC: Chinese Center for Disease Control and Prevention
